# Late-life difficult-to-treat depression and dementia subtypes: a naturalistic cohort study using electronic health records

**DOI:** 10.3389/fpsyt.2026.1795874

**Published:** 2026-05-04

**Authors:** Beatriz Pozuelo Moyano, Vasiliki Orgeta, Armin von Gunten, Pierre Vandel, Ruimin Ma, Robert Stewart, Christoph Mueller

**Affiliations:** 1 Service of Old Age Psychiatry, Department of Psychiatry, Lausanne University Hospital (CHUV) and University of Lausanne, Prilly, Switzerland; 2 Division of Psychiatry, University College London, London, United Kingdom; 3 King’s College London, Institute of Psychiatry, Psychology and Neuroscience, London, United Kingdom; 4 South London and Maudsley NHS Foundation Trust, London, United Kingdom

**Keywords:** Alzheimer, vascular dementia, dementia with lewy bodies, dementia subtypes, late-life depression, difficult-to-treat-depression

## Abstract

**Introduction:**

Late-life difficult-to-treat depression (LL-DTD) and dementia frequently coexist in later life, but it remains unclear whether clinical and sociodemographic characteristics, as well as medication exposure patterns, differ across dementia subtypes among older adults with both conditions.

**Methods:**

We analysed anonymised electronic health records from a south London catchment area. We included patients aged ≥60 years at first recorded depression diagnosis with a dementia diagnosis. LL-DTD was defined as inadequate response to ≥2 antidepressant trials. Dementia diagnoses were classified as Alzheimer’s disease (AD), vascular dementia (VD), mixed AD/VD, dementia with Lewy bodies (DLB), or other/unspecified dementia. Around the index depressive episode, we captured sociodemographics, depressive symptoms, physical comorbidity, and medication indicators. We used multivariate logistic regression to examine cross-sectional correlates distinguishing dementia subtypes (with AD as reference) within the LL-DTD and dementia sample. We conducted stratified analyses comparing non-AD versus AD dementia by the temporal order of dementia and depression diagnoses.

**Results:**

Among 890 older adults with LL-DTD and dementia, AD was the most common subtype (33.9%), followed by mixed AD/VD (22.5%), VD (22.2%), other/unspecified dementia (15.2%), and DLB (6.2%). Depressive symptom profiles and psychotropic treatment history were broadly similar across subtypes. Compared with AD, VD was associated with greater functional impairment, while greater physical comorbidity burden was more evident in VD and mixed AD/VD.

**Discussion:**

Somatic multimorbidity and functional impairment provided the clearest clinical separation between subgroups, while depressive symptom patterns and medication exposure appeared largely non-specific across dementia subtypes. This underscores the importance of multimorbidity and physical health burden in understanding heterogeneity of dementia outcomes in LL-DTD.

## 1 Introduction

Late-life depression (LLD) is among the most common psychiatric disorders in older adults and is often characterised by a chronic course, high recurrence, and substantial functional impairment (1). Beyond its psychiatric burden, LLD is consistently associated with an increased risk of subsequent dementia, including Alzheimer’s disease (AD) and vascular dementia (VD) (2). Evidence also indicates that LLD is associated with a high burden of medical comorbidity, particularly cardiovascular and cerebrovascular disease, which in turn has been linked to poorer treatment response (2). In addition, recent longitudinal studies have shown that major and subsyndromal depression are associated with an accelerated accumulation of somatic multimorbidity over time (3).

A major and growing clinical challenge within this population is difficult-to-treat depression in late-life (LL-DTD), which has been linked to poorer long-term outcomes, including faster cognitive decline and higher dementia risk, and is frequently accompanied by somatic multimorbidity and cardiovascular factors that may contribute to neurodegenerative and cerebrovascular processes (4–7).

Depression, and particularly LL-DTD, is not only associated with an increased risk of dementia but is also a common clinical syndrome within established dementia. Review evidence indicates that individuals with dementia are twice as likely as age-matched controls to develop major depressive disorders, and that up to one-third of people with all-cause dementia develop depression (8). In addition, depression in dementia often shows limited response to antidepressant treatment (8).

Depression and dementia are clinically heterogeneous, and there is growing interest in symptom-domain approaches rather than treating it as a unitary syndrome (9). Evidence suggests that the midlife depression and dementia association is not uniform across the different depression symptom profiles (10). In longitudinal cohort studies, the association appears to be driven by specific symptom domains, including low self-confidence, cognitive and executive dysfunction, impaired social connection, and anxiety-related features (10). Furthermore, given the differing aetiologies of dementia subtypes, examining depressive features across subtypes may provide an additional way to characterise this heterogeneity. However, most studies have focused on AD and VD, leaving the clinical profile of LL-DTD in other syndromes, such as dementia with Lewy bodies (DLB), comparatively underexplored (7). It therefore remains unclear which depressive symptom features and pharmacological treatments are most closely associated with different dementia subtypes among patients with LL-DTD. Clarifying these differential associations may improve clinical characterisation and subtype-oriented assessment within this comorbid population.

In previous work, we have observed that LL-DTD is associated with a greater burden of somatic comorbidity (5). However, beyond cardiovascular disease, the distribution of broader somatic comorbidities and multimorbidity across different dementia subtypes in LL-DTD remains insufficiently characterised. There is also a lack of comparative evidence on treatment patterns involving other medication classes used for augmentation, such as antipsychotics or mood stabilisers, as well as on the cumulative number of treatment attempts across dementia subtypes.

Using a large UK mental healthcare electronic health record resource, we investigated older adults with LL-DTD and comorbid dementia to describe and compare sociodemographic characteristics, depressive symptom features, functional and physical health comorbidity, as well as psychotropic medication indicators, across dementia subtypes (AD, VD, mixed AD/VD, DLB, and other/unspecified dementia), and to quantify subtype differences between AD and the other dementia subtypes. Because dementia and depression diagnoses may occur in different temporal sequences in routine clinical data, reflecting distinct clinical pathways (e.g., neuropsychiatric manifestations of dementia versus depression preceding later neurodegenerative evolution), we also examined whether the main patterns differed when analyses were stratified by temporal order of dementia and depression diagnoses.

## 2 Materials and methods

### 2.1 Data source

We conducted the study using de-identified electronic health records from the South London and Maudsley NHS Foundation Trust (SLaM) (11, 12), accessed via the Clinical Record Interactive Search (CRIS) platform. CRIS provides research access to de-identified data from more than 400,000 health records from SLaM within a robust governance framework. CRIS has ethical approval as an anonymised data resource (Oxford Research Ethics Committee C; 23/SC/0257).

The data of interest was extracted from structured fields in the source record or from free-text clinical documents using natural language processing (NLP) algorithms and the General Architecture for Text Engineering (GATE) software (11, 13).

### 2.2 Sample

The present analyses focused on the subgroup meeting criteria for LL-DTD and with a recorded diagnosis of dementia. Using the CRIS platform we identified patients aged ≥ 60 years with a recorded diagnosis of depressive episode or recurrent depressive disorder (according to ICD-10, using F32/F33 criteria (14)) made by SLaM services between 1st January 2008 and 31st December 2023. The index date was defined as the first recorded depressive disorder diagnosis at age ≥ 60 years.

### 2.3 Definition of difficult to treat late-life depression and dementia

LL-DTD was defined as a depressive disorder in individuals aged ≥60 years with evidence of inadequate response to at least two antidepressant trials, operationalised as prescriptions for two or more different antidepressant medications recorded in the mental health record (from 6 months before first LLD diagnosis to the end of their record). We also used a pre-established, machine learning-based NLP approach to identify clinician statements indicating treatment-resistant depression (15). Patients were considered to have underlying dementia if an ICD-10 diagnosis of F00–F03 or a free-text diagnosis of DLB (16) was recorded at any point in their electronic health record, either before or after the depression diagnosis. For subtype analyses, dementia was classified into five categories: (1) AD, (2) VD, (3) mixed AD/VD, (4) dementia with DLB, and (5) other/unspecified dementia if none of the aforementioned diagnoses was present. Dementia subtype was assigned using diagnoses recorded at any time in the patient record: DLB if any DLB diagnosis was present; mixed-type if F00.2 or both F00 and F01 were recorded; AD if only F00 was recorded; VD if only F01 was recorded; and unspecified/other if none of these diagnoses were present.

### 2.4 Measures

Sociodemographic variables included age, sex, ethnicity (White, Black, Asian, Other), marital/cohabitation status, and neighbourhood-level deprivation measured using the Index of Multiple Deprivation (IMD) (17). The Health of the Nation Outcome Scales (HoNOS (18)) was used to derive indicators of mental health symptoms and functioning (ie. depressed mood, activities of daily living (ADLs), and physical illness/problems). This is a well-established and routinely used scale in UK and European mental health services to measure patient wellbeing. Each subscale is rated from 0 (no problem) to 4 (severe or very severe problem). We dichotomised the relevant subscales (see below) to identify patients with difficulties (scoring 2–4) versus those without or with only minor problems (scoring 0–1) (16), and we used the HoNOS score closest to the index date of first depression diagnosis above the age of 60 years. We further employed NLP algorithms to identify recording of depressive symptoms and contextual factors in the patient’s free-text record (clinical events, correspondence) in a one-year window around first depression diagnosis (ie. from 6 months before to 6 months after the first recorded depression diagnosis at age ≥60 years) (11, 19). Depressive symptoms were aligned with ICD-10 and ICD-11 (including the feature of hopelessness) and retrieved from HoNOS to measure depressed mood and agitated behaviour and through NLP to detect anhedonia/poor motivation, anergia/low energy, worthlessness, guilt feelings, hopelessness, suicidal ideation, poor concentration, disturbed sleep, lack of appetite/weight loss (20, 21). Additional contextual variables captured were apathy and anxiety through NLP and problems with activities of daily living (ADLs) from HoNOS.

The medication NLP algorithm, which was applied to ascertain number of antidepressant trials across the electronic mental health record, was also used to identify types of antidepressants (selective serotonin reuptake inhibitor (SSRI), serotonin–noradrenaline reuptake inhibitor (SNRI), mirtazapine, tricyclic antidepressants), as well as antipsychotics and mood stabilisers, in a window from 6 months before to 6 months after index date as a proxy for prescribing at first depression diagnosis at age ≥60 (16). The NLP tools used in this study were existing CRIS applications implemented within the established CRIS NLP pipeline (see https://www.maudsleybrc.nihr.ac.uk/facilities/clinical-record-interactive-search-cris/cris-natural-language-processing/nlp-applications-library/).

Physical health was both ascertained through the HoNOS Physical Illness and/or Disability Scale (classified as minor, mild, and moderate/severe). Additionally, physical health conditions were identified from the full mental health record using a suite of MedCAT NLP algorithms (22, 23). The 21 conditions were: arthritis, asthma, atrial fibrillation, cerebrovascular accident, chronic kidney disease, chronic liver disease, chronic obstructive pulmonary disease, chronic sinusitis, coronary arteriosclerosis, diabetes mellitus, eczema, epilepsy, heart failure, systemic arterial hypertension, inflammatory bowel disease, ischaemic heart disease, migraine, multiple sclerosis, myocardial infarction, Parkinson’s disease, psoriasis, and transient ischaemic attack. For descriptive tables and regression analyses, we reported only individual physical health conditions with an overall prevalence of ≥10% in the cohort to avoid sparse estimates; however, the physical comorbidity score was derived using the full set of conditions (including those with <10% prevalence).

### 2.5 Statistical analysis

Patient characteristics were summarized across dementia subtypes (AD, VD, mixed AD/VD, DLB, and other/unspecified dementia) (**Table 1**). Group differences were assessed using ANOVA for continuous variables and chi-square tests for categorical variables. To quantify subtype differences relative to AD, we fitted separate logistic regression models for each patient characteristic with AD as the reference category and reported odds ratios (ORs) with 95% confidence intervals for each patient characteristic, adjusting for age, sex, ethnicity, marital/cohabitation status and neighbourhood-level deprivation. Therefore, the estimates presented in **Table 2** represent associations adjusted for sociodemographic covariates rather than a single fully multivariable model including all clinical predictors simultaneously. This approach was used to provide comparable effect estimates for individual clinical features across dementia subtypes. Continuous patient characteristics (age, deprivation index, and comorbidity score) were dichotomised by creating binary variables indicating whether each value was above the median for the full cohort. As complementary analyses, age, deprivation score, and comorbidity score were also examined as continuous (dependent) variables fitting adjusted linear regression models.

**Table 1:** Characteristics of patients with LL-DTD and dementia, by dementia subtype (n=890).

Variable	Alzheimer’s disease (n=302)	Vascular dementia (n=198)	Mixed AD & vascular dementia (n=200)	Dementia with Lewy bodies (n=55)	Other/unspecified dementia (n=135)	p-value ^a^
Sociodemographic characteristics ^b^						
Age, mean (SD), years	77.5 (8.6)	78.1 (8.1)	78.4 (7.9)	75.7 (7.8)	77.1 (8.1)	0.192
Female sex	73.5%	60.6%	67.5%	63.6%	54.8%	0.001*
Ethnicity						0.315
White	73.1%	71.9%	73.2%	75.9%	80.9%	
Black	12.5%	18.8%	15.2%	16.7%	6.9%	
Asian	9.8%	6.2%	7.6%	5.6%	7.6%	
Other	4.7%	3.1%	4.0%	1.9%	4.6%	
Married/cohabiting	28.9%	31.2%	37.3%	35.4%	38.4%	0.230
Index of Multiple Deprivation (IMD), mean (SD)	23.78 (9.76)	25.47 (9.81)	25.07 (9.87)	25.90 (10.61)	24.33 (9.89)	0.282
Clinical features at/around index depression diagnosis (HoNOS) ^b,c^						
Problems with activities of daily living	46.8%	68.4%	55.9%	50.9%	58.9%	<0.001*
Physical comorbidity count, mean (SD)	1.76 (1.28)	2.70 (1.52)	2.46 (1.31)	2.11 (1.36)	1.76 (1.35)	<0.001*
Physical health problems (HoNOS physical illness scale)						<0.001*
Physical illness minor (score 0-1)	48.0%	23.6%	32.3%	34.0%	27.9%	
Physical illness mild (score 2)	29.9%	33.3%	30.8%	28.3%	27.1%	
Physical illness moderate/severe (score 3–4)	22.1%	43.1%	36.9%	37.7%	45.0%	
Physical health conditions from MedCat^d^						
Hypertension	64.8%	83.8%	81.0%	72.7%	60.0%	<0.001*
Diabetes mellitus	44.4%	49.5%	51.5%	41.8%	40.7%	0.235
Cerebrovascular accident (stroke)	19.5%	58.6%	46.0%	30.9%	24.4%	<0.001*
Arthritis	33.8%	27.8%	38.5%	34.5%	26.7%	0.102
Chronic kidney disease	18.2%	17.2%	25.5%	30.9%	23.0%	0.058
Myocardial infarction	12.6%	23.2%	21.5%	23.6%	14.8%	0.009*
Chronic obstructive pulmonary disease	16.3%	16.2%	20.0%	23.6%	18.5%	0.583
Ischaemic heart disease	13.9%	23.2%	21.0%	16.4%	14.1%	0.042
Asthma	15.2%	16.2%	12.0%	18.2%	12.6%	0.631
Transient ischaemic attack	7.9%	23.7%	18.0%	9.1%	8.9%	<0.001*
Heart failure	11.6%	17.2%	12.0%	20.0%	9.6%	0.116
Atrial fibrillation	7.6%	16.2%	16.5%	7.3%	14.1%	0.009*
Parkinson’s disease	4.6%	4.5%	8.0%	32.7%	31.1%	<0.001*
Depressive symptoms profiles						
Depressed mood score, %	64.3%	73.3%	67.2%	67.9%	73.6%	0.179
Anhedonia/poor motivation^d^	29.8%	34.3%	28.5%	25.5%	41.5%	0.062
Anergia/low energy^d^	42.1%	35.9%	36.0%	29.1%	37.0%	0.317
Worthlessness	9.3%	11.1%	9.5%	14.5%	11.1%	0.775
Guilt feelings	30.5%	26.3%	30.0%	30.9%	23.0%	0.483
Hopelessness	33.1%	31.3%	32.5%	32.7%	39.3%	0.634
Suicidal ideation	34.1%	28.8%	37.5%	40.0%	37.0%	0.309
Poor concentration	43.4%	38.4%	42.0%	40.0%	41.5%	0.861
Agitated behaviour problems	11.9%	19.5%	19.0%	20.8%	18.6%	0.107
Disturbed sleep	69.5%	73.7%	72.5%	78.2%	70.4%	0.659
Lack of appetite/weight loss	53.0%	54.0%	51.5%	60.0%	61.5%	0.361
Other symptoms/contextual factors						
Anxiety	84.4%	81.3%	81.5%	78.2%	80.7%	0.735
Apathy	12.6%	16.2%	11.5%	20.0%	11.9%	0.353
Medications						
≥3 antidepressant trials recorded	39.7%	38.4%	40.0%	36.4%	37.0%	0.968
Selective serotonin reuptake inhibitor (SSRI)	77.2%	83.3%	84.0%	83.6%	81.5%	0.281
Serotonin–norepinephrine reuptake inhibitor (SNRI)	34.8%	31.3%	36.0%	30.9%	33.3%	0.857
Mirtazapine	76.8%	73.7%	78.0%	76.4%	80.0%	0.738
Tricyclic antidepressant (TCA)	25.8%	29.8%	19.5%	21.8%	24.4%	0.192
Antipsychotic	38.7%	41.4%	40.0%	60.0%	42.2%	0.063
Mood stabiliser	9.3%	15.2%	12.5%	16.4%	4%	0.100

* and bold = p-value <0.05 after Benjamini-Hochberg correction; ^a^ t-test or chi^2^ test; ^b^ at or around the time of first late-life depression diagnosis; ^c^ Health of the Nation Outcome Scale (HoNOS) subscale scores 0-4 (0=least severe, 4 = most severe status) were categorised into scores of 0–1 representing that the patient was not experiencing problems in that domain, and scores of 2–4 representing experiencing problems; ^d^ Physical health conditions identified across the whole patients medical records using natural language processing.

**Table 2:** Associations of different dementia subtypes with LL-DTD characteristics (in adjusted logistic regression models reporting Odds ratios; AD as comparison group).

Variable	Vascular vs AD^a^	Mixed vs AD^a^	DLB vs AD^a^	Other vs AD^a^
Sociodemographic characteristics^b^				
Age above median (years)	1.25 (0.84–1.84)	1.48 (1.00–2.18)	0.65 (0.33–1.27)	1.16 (0.74–1.81)
Female sex	0.52 (0.34–0.78)*	0.73 (0.48–1.12)	0.61 (0.32–1.18)	0.47 (0.30–0.75)*
White ethnicity	0.93 (0.61–1.42)	1.05 (0.68–1.62)	1.17 (0.57–2.41)	1.69 (0.99–2.91)
Black ethnicity	1.71 (1.00–2.91)	1.29 (0.74–2.24)	1.29 (0.54–3.07)	0.51 (0.23–1.15)
Asian ethnicity	0.57 (0.27–1.20)	0.65 (0.32–1.32)	0.58 (0.17–2.04)	0.56 (0.24–1.30)
Other ethnicity	0.83 (0.30–2.31)	0.97 (0.36–2.56)	0.57 (0.07–4.54)	1.12 (0.38–3.34)
Married/cohabiting	1.09 (0.71–1.67)	1.56 (1.03–2.35)	1.36 (0.70–2.65)	1.41 (0.88–2.24)
Deprivation above median (IMD)	1.34 (0.91–1.96)	1.45 (0.99–2.13)	1.53 (0.81–2.88)	1.00 (0.65–1.55)
Clinical features at/around index depression diagnosis (HoNOS) ^b,c^				
Problems with activities of daily living	2.24 (1.49–3.35)*	1.28 (0.87–1.88)	1.38 (0.73–2.61)	1.55 (0.99–2.43)
Physical comorbidity score >3 (vs ≤3)	2.28 (1.54–3.36)*	2.43 (1.65–3.59)*	1.59 (0.85–3.00)	1.10 (0.70–1.74)
Physical illness minor	0.35 (0.23–0.53)*	0.54 (0.37–0.81)*	0.49 (0.25–0.96)	0.39 (0.24–0.62)*
Physical illness mild	1.22 (0.81–1.85)	1.15 (0.76–1.74)	0.92 (0.45–1.87)	1.02 (0.63–1.66)
Physical illness moderate/severe	2.59 (1.71–3.93)*	1.86 (1.22–2.84)*	2.47 (1.28–4.78)	2.55 (1.59–4.09)*
ICD-10/11 depressive symptoms				
Depressed mood	1.55 (1.01–2.35)	1.17 (0.78–1.76)	1.42 (0.71–2.85)	1.56 (0.96–2.55)
Anhedonia/poor motivation	1.17 (0.78–1.76)	0.95 (0.63–1.43)	0.68 (0.33–1.41)	1.62 (1.04–2.54)
Anergia/low energy	0.79 (0.54–1.17)	0.79 (0.53–1.17)	0.65 (0.33–1.27)	0.84 (0.54–1.31)
Worthlessness	1.48 (0.79–2.76)	1.26 (0.66–2.40)	2.16 (0.90–5.21)	1.36 (0.67–2.76)
Guilt feelings	0.95 (0.63–1.46)	1.10 (0.72–1.66)	0.99 (0.50–1.97)	0.74 (0.45–1.22)
Hopelessness	0.90 (0.60–1.35)	1.02 (0.68–1.53)	1.15 (0.60–2.22)	1.31 (0.83–2.05)
Suicidal ideation	0.84 (0.56–1.27)	1.20 (0.81–1.79)	1.53 (0.81–2.89)	1.19 (0.76–1.88)
Poor concentration	0.80 (0.54–1.17)	0.88 (0.60–1.29)	0.91 (0.49–1.71)	0.85 (0.55–1.31)
Agitation	1.88 (1.11–3.17)	1.70 (1.00–2.89)	2.14 (0.96–4.77)	1.66 (0.90–3.06)
Disturbed sleep	1.28 (0.84–1.95)	1.28 (0.84–1.95)	1.70 (0.80–3.60)	1.02 (0.64–1.64)
Lack of appetite/weight loss	1.17 (0.80–1.71)	1.08 (0.74–1.58)	1.44 (0.77–2.71)	1.51 (0.97–2.35)
Physical health condition from MedCat^d^				
Hypertension	2.82 (1.75–4.52)*	2.51 (1.57–3.99)*	1.47 (0.73–2.96)	0.82 (0.52–1.28)
Diabetes mellitus	1.31 (0.89–1.95)	1.35 (0.91–2.00)	0.78 (0.40–1.51)	0.90 (0.57–1.42)
Cerebrovascular accident (stroke)	5.81 (3.80–8.89)*	3.42 (2.25–5.21)*	1.61 (0.80–3.24)	1.43 (0.86–2.37)
Arthritis	0.82 (0.54–1.25)	1.19 (0.80–1.78)	1.03 (0.53–2.00)	0.83 (0.51–1.34)
Chronic kidney disease	0.85 (0.52–1.40)	1.64 (1.05–2.58)	1.65 (0.81–3.37)	1.19 (0.69–2.04)
Myocardial infarction	1.78 (1.09–2.89)	1.69 (1.03–2.77)	1.99 (0.94–4.20)	1.12 (0.62–2.04)
Chronic obstructive pulmonary disease	1.02 (0.61–1.69)	1.31 (0.80–2.14)	1.70 (0.82–3.53)	1.14 (0.65–2.00)
Ischaemic heart disease	1.65 (1.02–2.67)	1.53 (0.94–2.50)	0.96 (0.40–2.30)	0.87 (0.47–1.62)
Asthma	1.11 (0.66–1.87)	0.82 (0.47–1.43)	1.26 (0.56–2.84)	0.93 (0.50–1.72)
Transient ischaemic attack	3.61 (2.07–6.31)*	2.73 (1.53–4.85)*	1.37 (0.49–3.82)	1.03 (0.47–2.25)
Heart failure	1.58 (0.93–2.70)	0.92 (0.51–1.67)	2.45 (1.12–5.38)	0.76 (0.36–1.57)
Atrial fibrillation	2.05 (1.12–3.75)	2.13 (1.17–3.89)	0.79 (0.22–2.81)	1.90 (0.96–3.75)
Parkinson’s disease	0.92 (0.36–2.33)	1.82 (0.82–4.03)	10.54 (4.50–24.72)*	8.60 (4.24–17.44)*
Other symptoms/contextual factors				
Anxiety	0.65 (0.38–1.10)	0.80 (0.46–1.39)	0.61 (0.27–1.38)	0.65 (0.36–1.19)
Apathy	1.33 (0.78–2.29)	0.90 (0.50–1.62)	1.62 (0.71–3.67)	0.88 (0.45–1.72)
Medications				
≥3 antidepressant trials recorded	0.98 (0.66–1.46)	1.05 (0.71–1.56)	0.80 (0.41–1.54)	0.79 (0.50–1.25)
SSRI	1.55 (0.95–2.51)	1.65 (1.01–2.68)	1.57 (0.69–3.56)	1.44 (0.83–2.49)
SNRI	0.91 (0.60–1.36)	1.07 (0.71–1.59)	0.76 (0.39–1.51)	0.88 (0.56–1.40)
Mirtazapine	0.81 (0.52–1.26)	0.98 (0.63–1.54)	0.90 (0.44–1.85)	1.14 (0.67–1.93)
TCA	1.25 (0.82–1.90)	0.69 (0.43–1.09)	0.81 (0.39–1.70)	0.96 (0.58–1.58)
Antipsychotic	1.12 (0.76–1.66)	1.12 (0.76–1.65)	2.52 (1.31–4.85)	1.10 (0.71–1.72)
Mood stabiliser	1.87 (1.06–3.33)	1.45 (0.79–2.63)	1.47 (0.59–3.66)	0.73 (0.34–1.59)

* and bold = p-value <0.05 after Benjamini-Hochberg correction; ^a^ Adjusted model = adjusted for age, sex, ethnicity, marital status and deprivation; ^b^ at or around the time of first late-life depression diagnosis; ^c^ Health of the Nation Outcome Scale (HoNOS) subscale scores 0-4 (0=least severe, 4 = most severe status) were categorised into scores of 0–1 representing that the patient was not experiencing problems in that domain, and scores of 2–4 representing experiencing problems; ^d^ Physical health conditions identified across the whole patients medical records using natural language processing.

To explore whether the temporal ordering of dementia and depression diagnoses influenced the observed associations, we conducted additional sensitivity analyses using a binary dementia grouping (non-AD vs AD). For these analyses, AD and mixed AD/VD dementia were combined into a single AD group, and all remaining subtypes (vascular dementia, DLB, and other/unspecified dementia) were grouped as non-AD. We then estimated logistic regression models in (i) the full sample and (ii) two strata defined by temporal order of diagnoses: dementia diagnosis before depression diagnosis or up to 3 months after the index depression diagnosis and dementia diagnosis at least 3 months after the depression diagnosis (5, 24).

All analyses were corrected for multiple comparisons using the Benjamini–Hochberg false discovery rate (FDR) procedure (25). P-values were ranked from smallest to largest, and adjusted p-values were calculated as *p × (m/i)*, where *i* is the rank and *m* the total number of tests. Results were considered statistically significant at FDR-adjusted p < 0.05.

Missingness was below 9% for individual variables and 89% of included patients had complete data. The main sources of missing data were marital status (6% had only marital status missing) and HoNOS subscales (2% had only HoNOS subscale missing). Given the low overall level of missingness, a complete-case analysis was considered appropriate and did not materially affect the descriptive distributions.

## 3 Results

### 3.1 Sample characteristics and dementia subtype distribution

The analytic sample comprised 890 patients with LL-DTD and dementia. Dementia subtypes were AD (n=302, 33.9%), VD (n=198, 22.2%), mixed AD/VD (n=200, 22.5%), DLB (n=55, 6.2%), and other/unspecified dementia (n=135, 15.2%).

### 3.2 Characteristics of patients with LL-DTD and dementia

Patient characteristics by dementia subtype are presented in **Table 1**. Overall differences across dementia subtypes were observed for sex, functional impairment, and multiple indicators of physical health burden. The proportion of women was highest in AD (73.5%) and lowest in other/unspecified dementia (54.8%). Problems with activities of daily living were most common in VD (68.4%) and least common in AD (46.8%). Markers of somatic burden also varied: mean physical comorbidity count was highest in VD (2.70, SD 1.52) and lowest in AD (1.76, SD 1.28) and other/unspecified dementia (1.76, SD 1.35), while moderate/severe physical illness on the HoNOS physical illness scale was most frequent in other/unspecified dementia (45.0%) and least frequent in AD (22.1%).

Among individual physical health conditions meeting the ≥10% prevalence threshold, several cardio-cerebrovascular conditions differed across subtypes, with the highest prevalences generally observed in VD. Hypertension was most common in VD (83.8%) and least common in other/unspecified dementia (60.0%); stroke was most common in VD (58.6%) and least common in AD (19.5%); myocardial infarction was most common in DLB (23.6%) and least common in AD (12.6%); and transient ischaemic attack was most common in VD (23.7%) and least common in AD (7.9%). Atrial fibrillation also varied, being most frequent in mixed AD/VD (16.5%) and least frequent in DLB (7.3%). Finally, Parkinson’s disease showed the largest subtype variation, being far more prevalent in DLB (32.7%) and other/unspecified dementia (31.1%) than AD (4.6%).

### 3.3 Associations between characteristics of patients and the different dementia subtypes compared to Alzheimer’s disease

Associations of baseline characteristics with dementia subtype (with AD as the reference) are shown in **Table 2**, based on models adjusted for age, sex, ethnicity, marital/cohabitation status and neighbourhood deprivation. Overall, subtype differences were driven chiefly by somatic and functional burden, particularly vascular morbidity, with fewer differences in depressive symptom indicators and medication history. Vascular dementia was characterised by lower odds of female sex and greater functional and physical burden (including ADL problems, comorbidity score >3, and moderate/severe physical illness), alongside a strong cardio-cerebrovascular profile (notably hypertension, stroke and transient ischaemic attack (TIA)). Mixed AD/VD showed a broadly similar somatic/vascular pattern (including comorbidity score >3, moderate/severe physical illness, hypertension, stroke and TIA). DLB was distinguished by a greater association with Parkinson’s disease. Other/unspecified dementia was less likely to be female and showed greater physical illness burden, and also a strong association with Parkinson’s disease.

When modelling age at depression diagnosis, index of multiple deprivations and the physical comorbidity score as continuous variables the following significant differences emerged in adjusted linear regression models: The deprivation score was 1.92 (95% confidence interval (CI): 0.09-3.75; p=0.040) points higher in mixed dementia than in AD. Compared to AD, the co-morbidity score was 1.11 (95% CI: 0.71-1.52; p<0.001) points higher in vascular dementia, 1.07 (95% CI: 0.67-1.47; p<0.001) points higher in mixed dementia, and 0.89 (95% CI: 0.22-1.56; p=0.010) points higher in DLB. No significant differences between AD and other dementia subtypes were detected in relation to age at depression diagnosis in linear regression models.

### 3.4 Temporal-order stratified analyses (non-AD vs AD)

In the full sample, overall differences between non-AD and AD dementia were observed for sex, problems with activities of daily living, minor and moderate/severe physical illness, cerebrovascular accident and Parkinson’s disease (see **Table 3**). Very similar associations were observed in the larger subgroup in which dementia was diagnosed at least 3 months after depression (n = 534; 40.3% non-AD dementia). The direction of associations was also largely consistent in the smaller subgroup where dementia was diagnosed before (or up to 3 months after) the depression diagnosis (n = 356; 48.6% non-AD dementia), although fewer associations reached statistical significance.

**Table 3:** Associations of Non-AD dementia (compared to AD) with different LL_DTD characteristics in the full sample and by temporal order of the dementia diagnosis (in adjusted logistic regression models reporting Odds ratios).

Variable	Non-AD vs AD (full sample; cohort n=890)^a^	Non-AD vs AD (dementia diagnosis before depression diagnosis or up to 3 months after; n=356)^a^	Non-AD vs AD (dementia diagnosis at least 3 months after depression diagnosis; n=534)^a^
Sociodemographic characteristics^b^			
Age above median (years)	0.95 (0.71-1.27)	0.89 (0.56-1.42)	0.91 (0.62-1.34)
Female sex	0.58 (0.43-0.79)*	0.57 (0.34-0.94)	0.59 (0.40-0.86)*
White ethnicity	1.13 (0.82-1.57)	1.58 (0.95-2.62)	0.93 (0.60-1.42)
Black ethnicity	1.08 (0.72-1.63)	0.94 (0.49-1.80)	1.17 (0.68-1.99)
Asian ethnicity	0.67 (0.39-1.17)	0.44 (0.19-1.03)	0.90 (0.43-1.86)
Other ethnicity	0.91 (0.43-1.92)	0.60 (0.19-1.85)	1.25 (0.45-3.46)
Married/cohabiting	1.02 (0.75-1.38)	0.93 (0.57-1.51)	1.06 (0.72-1.58)
Deprivation above median (IMD)	1.06 (0.80-1.40)	0.99 (0.63-1.55)	1.10 (0.76-1.60)
Clinical features at/around index depression diagnosis (HoNOS) ^b,c^			
Problems with activities of daily living	1.66 (1.24-2.23)*	1.02 (0.61-1.69)	2.03 (1.39-2.96)*
Physical comorbidity score >3 (vs ≤3)	1.17 (0.88-1.55)	1.19 (0.75-1.89)	1.17 (0.81-1.70)
Physical illness minor	0.48 (0.35-0.66)*	0.60 (0.37-0.99)	0.42 (0.28-0.63)*
Physical illness mild	1.05 (0.77-1.43)	0.81 (0.49-1.35)	1.22 (0.82-1.81)
Physical illness moderate/severe	1.93 (1.43-2.61)*	1.86 (1.16-2.98)	2.00 (1.34-2.99)*
ICD-10/11 depressive symptoms			
Depressed mood	1.44 (1.05-1.97)	1.43 (0.88-2.32)	1.50 (0.98-2.28)
Anhedonia/poor motivation	1.26 (0.94-1.70)	1.55 (0.94-2.56)	1.12 (0.77-1.64)
Anergia/low energy	0.87 (0.65-1.16)	1.02 (0.63-1.64)	0.82 (0.57-1.19)
Worthlessness	1.38 (0.88-2.17)	2.90 (1.16-7.26)	1.07 (0.61-1.87)
Guilt	0.85 (0.62-1.16)	1.50 (0.88-2.58)	0.63 (0.42-0.94)
Hopelessness	1.05 (0.78-1.41)	1.05 (0.64-1.73)	1.08 (0.74-1.58)
Suicidality	0.96 (0.71-1.29)	0.71 (0.44-1.16)	1.12 (0.76-1.64)
Poor concentration	0.87 (0.66-1.16)	0.85 (0.53-1.36)	0.91 (0.63-1.31)
Agitation/problem	1.44 (0.99-2.10)	1.20 (0.70-2.06)	1.63 (0.95-2.80)
Disturbed sleep	1.11 (0.81-1.52)	0.81 (0.49-1.34)	1.31 (0.87-1.99)
Lack of appetite//weight problem	1.27 (0.96-1.68)	1.33 (0.85-2.10)	1.22 (0.84-1.76)
Physical health condition from MedCat^d^			
Hypertension	1.12 (0.81-1.54)	1.32 (0.80-2.17)	1.05 (0.68-1.62)
Diabetes	0.95 (0.71-1.28)	0.84 (0.52-1.36)	1.09 (0.75-1.60)
Cerebrovascular accident (stroke)	1.76 (1.31-2.36)*	1.72 (1.07-2.76)	1.75 (1.19-2.58)*
Arthritis	0.79 (0.58-1.07)	0.85 (0.51-1.40)	0.80 (0.54-1.17)
Chronic kidney disease	0.85 (0.60-1.20)	1.12 (0.61-2.06)	0.76 (0.49-1.17)
Myocardial infarction	1.23 (0.86-1.76)	1.73 (0.98-3.07)	0.96 (0.60-1.53)
Chronic obstructive pulmonary disease	1.02 (0.71-1.47)	0.84 (0.47-1.53)	1.16 (0.73-1.86)
Ischemic heart disease	1.05 (0.73-1.51)	1.07 (0.58-1.94)	1.03 (0.65-1.65)
Asthma	1.15 (0.77-1.70)	0.89 (0.46-1.75)	1.39 (0.85-2.29)
Transient ischemic attack	1.40 (0.94-2.09)	1.75 (0.91-3.35)	1.20 (0.71-2.01)
Heart failure	1.43 (0.95-2.17)	1.43 (0.73-2.82)	1.52 (0.89-2.58)
Atrial fibrillation	1.25 (0.82-1.92)	1.10 (0.53-2.30)	1.46 (0.86-2.48)
Parkinson disease	3.19 (1.96-5.19)*	4.45 (1.68-11.78)*	3.00 (1.69-5.32)*
Other symptoms/contextual factors			
Anxiety	0.71 (0.48-1.05)	0.50 (0.28-0.90)	0.95 (0.56-1.62)
Apathy	1.26 (0.84-1.90)	1.18 (0.67-2.07)	1.12 (0.59-2.13)
Medications			
≥3 antidepressant trials recorded	0.87 (0.65-1.17)	0.83 (0.50-1.38)	0.96 (0.66-1.39)
SSRI	1.25 (0.87-1.79)	1.17 (0.68-2.00)	1.49 (0.90-2.47)
SNRI	0.86 (0.63-1.16)	0.80 (0.49-1.30)	0.90 (0.61-1.33)
Mirtazapine	0.93 (0.67-1.29)	0.85 (0.51-1.41)	0.98 (0.62-1.55)
TCA	1.25 (0.90-1.73)	1.59 (0.90-2.80)	1.16 (0.77-1.73)
Antipsychotic	1.18 (0.89-1.58)	0.98 (0.61-1.55)	1.32 (0.91-1.91)
Mood stabiliser	1.17 (0.76-1.81)	1.10 (0.48-2.54)	1.22 (0.72-2.04)

* and bold = p-value <0.05 after Benjamini-Hochberg correction; ^a^ Adjusted model = adjusted for age, sex, ethnicity, marital status and deprivation; ^b^ at or around the time of first late-life depression diagnosis; ^c^ Health of the Nation Outcome Scale (HoNOS) subscale scores 0-4 (0=least severe, 4 = most severe status) were categorised into scores of 0–1 representing that the patient was not experiencing problems in that domain, and scores of 2–4 representing experiencing problems; ^d^ Physical health conditions identified across the whole patients medical records using natural language processing.

## 4 Discussion

In this cross-sectional analysis of older adults with LL-DTD and comorbid dementia, we observed minimal differentiation by depressive symptom profiles and psychotropic treatment across dementia subtypes. In contrast, the most significant subtype differentiation was observed in physical multimorbidity, vascular/cardiometabolic burden, and functional impairment. Compared with AD, vascular dementia and mixed AD/VD were characterised by greater burden of activities-of-daily-living problems, higher physical comorbidity, more severe physical illness, and greater cerebrovascular and cardiometabolic morbidity.

Our findings complement prior longitudinal work linking LL-DTD to an increased risk of subsequent dementia and AD, particularly in late-onset depression (7). Whereas such longitudinal designs address whether LL-DTD predicts subsequent dementia, our analyses focus on how dementia subtypes differ within LL-DTD, by comparing sociodemographic, clinical (symptom, functional and comorbidity) and pharmacological treatment characteristics across dementia syndromes. To our knowledge, this is the first study to characterise depressive symptom profiles and detailed indicators of pharmacological treatment within LL-DTD across different dementia subtypes.

In contrast to recent midlife findings suggesting that dementia risk is driven by specific depressive symptom domains rather than depression as a unitary construct (10), we did not observe a remarkable symptom profile differentiating dementia subtypes within LL-DTD. This suggest that depressive symptom phenomenology alone offers limited discrimination between dementia subtypes in this comorbid population.

The findings suggest that broader physical multimorbidity burden may be an important correlate across dementia subtypes. Vascular dementia and mixed AD/VD were characterised by greater overall somatic burden than “pure” AD. Moderate/severe physical illness and cardiovascular-related conditions showed relatively consistent associations with vascular dementia and mixed AD/VD. This is consistent with wider evidence that cardiometabolic and neurovascular burden become increasingly relevant in later-life depression and dementia (26, 27). While this pattern is clinically plausible, it should be interpreted cautiously because it is partly aligned with the diagnostic definitions of vascular and mixed AD/VD dementia, introducing a degree of circularity in interpretation (28). Further studies should test whether broader somatic multimorbidity remains associated with subtype after excluding vascular events that may contribute directly to subtype classification (e.g., stroke and transient ischaemic attack). DLB and the “other” dementia category were characterised by a distinct clinical profile, with Parkinson’s disease showing a strong association with both subtypes relative to AD. This finding is clinically unsurprising and supports the clinical and diagnostic coherence of these subtype groupings, given that parkinsonism is a core feature of DLB and that the “other” category may plausibly include under-recognised cases of DLB as well as Parkinson’s disease dementia (29).

We did not observed statistically significant differences in psychotropic medication indicators across dementia subtypes. Given the relatively small size of some subgroups (particularly DLB) and the pragmatic nature of our medication exposure measures (based on recorded prescribing indicators), these analyses may have been underpowered or insufficiently sensitive to detect subtype-specific differences. The broadly similar prescribing patterns are consistent with prior literature indicating that antidepressant benefit in depression with dementia is often limited overall (8). One hypothetical mechanistic interpretation is that monoaminergic dysfunction across dementia syndromes may constrain antidepressant effects (8).

Clinically, features such as the pattern of depressive symptoms, past medication history, and individual physical symptoms are unlikely to reliably guide decisions about dementia subtypes in routine practice; except where symptoms are directly linked to a specific subtype (for example, vascular features in vascular dementia). Instead, our results suggest that greater clinical attention should be given to broader somatic multimorbidity and functional impairment, particularly when older adults with LL-DTD present with emerging cognitive symptoms. These findings support the value of multidisciplinary assessment in this complex comorbid population.

A key strength of this study is its large, socially and ethnically diverse sample drawn from routine UK mental healthcare, which enhances generalisability to everyday clinical practice beyond highly selected trial populations. Using a rich EHR resource, we were able to compare dementia subtypes within a clinically severe phenotype of LL-DTD. By integrating structured clinical measures (e.g., HoNOS) with NLP-derived depressive symptom features and MedCAT-derived physical comorbidity data, we provide a more granular characterisation than studies relying solely on diagnostic codes. Despite these strengths, our study has several limitations. First, the analyses were cross-sectional within the LL-DTD plus dementia subgroup and therefore cannot establish causality or clarify temporal ordering. Second, LL-DTD was operationalised using prescribing history (≥2 antidepressant trials), which serves as a proxy for DTD in routine EHR data and may introduce heterogeneity and misclassification. Our definition reflects real-world prescribing history, rather than confirmed pharmacological treatment resistance, and recorded treatment changes may reflect tolerability, adverse effects, prescribing preferences, clinical complexity, or other non-efficacy reasons. In addition, we could not determine dose adequacy, treatment duration, adherence, sequencing versus overlap of antidepressant trials, or the interval between treatment attempts. As this operational LL-DTD definition determined the inclusion of the cohort, this imprecision may affect the interpretability of comparisons of all dementia subtypes. Third, dementia subtype classification was based on a pragmatic combination of ICD-10 diagnostic codes and free-text extraction from routine clinical records, which may introduce diagnostic misclassification. This is particularly relevant for distinctions between AD, mixed AD/VD dementia, and DLB, where overlap in clinical presentation and variability in documentation or coding practices may affect subtype assignment. DLB may also be under-recorded in routine care, and the relatively small DLB subgroup in our cohort may partly reflect diagnostic and coding practices rather than true prevalence. Because dementia subtype is the primary grouping variable in this study, such misclassification may affect the interpretability of between-subtype comparisons. Finally, another limitation is the potential for false-positive findings due to the large number of statistical tests carried out. However, the risk of false-positive findings was mitigated through application of the Benjamini–Hochberg procedure to control the false discovery rate. Furthermore, the use of separate regression models for multiple predictors may have increased the number of comparisons and limited the interpretability of correlated clinical features. Accordingly, the reported associations should be interpreted with caution and confirmed in independent cohorts.

Future research should use longitudinal designs with repeated, standardised assessments to determine whether dementia subtypes in LL-DTD differ in depressive symptom trajectories over time. Studies should also characterise treatment pathways in more detail (capturing dose, duration, adherence, tolerability and reasons for switching/augmentation) to clarify whether subtype differences emerge when prescribing is measured more precisely than simple drug-class exposure.

In conclusion, our findings suggest that in older adults with LL-DTD and dementia, depressive symptom profile and psychotropic treatment history are of limited value for distinguishing dementia subtypes. The findings also support multidisciplinary management models for this comorbid population, where old age psychiatry, memory services, and medical teams jointly address depression, dementia, multimorbidity, and functional decline.

## Data Availability

The raw data supporting the conclusions of this article will be made available by the authors, without undue reservation.
